# The Efficacy of Intraoperative Esophagogastroduodenoscopy in Localizing Retroperitoneal Bowel Injury: A Case Report

**DOI:** 10.7759/cureus.54057

**Published:** 2024-02-12

**Authors:** Muzi Meng, Cesar Riera Gonzalez, Yonas Teklu, Jorge Mosquera Zavaleta, Ajit Singh

**Affiliations:** 1 School of Medicine, American University of the Caribbean, Cupecoy, SXM; 2 General Surgery, BronxCare Health System, Bronx, USA

**Keywords:** emergency exploratory laparotomy, exploratory laparotomy, trauma, retroperitoneal bowel injury, duodenal hematoma, intraoperative egd

## Abstract

The efficacy of intraoperative esophagogastroduodenoscopy (EGD) in visualizing a patient’s small bowel interior to detect injuries or lesions, or conduct a leak test post-bowel anastomosis, makes it a preferred option among surgeons. However, it is not always available, can carry a risk of morbidity and mortality, or can prolong operative time if not performed by a proficient team. A 21-year-old male patient came to the emergency department with four gunshot wounds to his abdomen, with two on either side of the abdomen. Exploratory laparotomy was performed and through and through injuries were identified in the small bowel and at the junction of the third/fourth portion of the duodenum. It was challenging to gather the patient’s past medical history, particularly gastrointestinal bleeding history, due to the underlying medical condition. However, the patient had experienced a retroperitoneal bowel injury in the setting of duodenal hematoma that was not immediately identified at first glance. In this context, intraoperative endoscopy could be a significant adjunct to detect retroperitoneal bowel injury if rapidly available and in a controlled scenario. Moreover, the advantages of intraoperative EGD increase with positive collaboration between a general surgeon and a gastroenterologist.

## Introduction

The application of esophagogastroduodenoscopy (EGD) in exploring the stomach and retroperitoneal small bowel (duodenum) is common among surgeons. The procedure facilitates surgeons in identifying the site of retroperitoneal bowel injury. The effectiveness of intraoperative EGD in visualizing a patient’s small bowel interior makes it preferable in the setting of complex procedures. However, there is a risk of morbidity and mortality that cannot be ignored.

In this report, we review the case of a 21-year-old male who was admitted to the emergency department (ED) after receiving four gunshots. In this context, the surgeon performed an intraoperative EGD to localize the location of retroperitoneal bowel injury in the setting of duodenal hematoma, followed by primary resection and anastomosis surgery. The intraoperative EGD identified the location of the retroperitoneal bowel injury without the need to perform a Kocher maneuver for further exposure.

## Case presentation

A 21-year-old male patient came to the ED with four gunshot wounds to his abdomen, with two on either side of the abdomen. He was in hemorrhagic shock on admission, with a blood pressure of 70/40 mmHg and a heart rate of 100 beats/minute. Focused assessment with sonography for trauma was positive. His past medical history, past surgical history, and tobacco use status were unknown due to his underlying medical condition. A massive transfusion protocol was initiated, and the patient received four units of packed red blood cells (PRBCs) before surgery.

**Figure 1:**
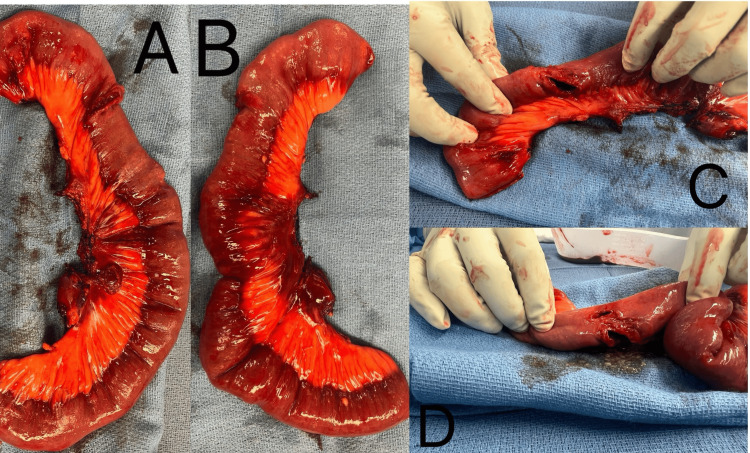
Through and through penetrating injuries of the small bowel. A and B: small bowel and associated mesentery resection. C and D: through and through penetrating injuries.

The patient was taken to the operating room for emergent exploratory laparotomy and identification of the source of uncontrolled bleeding. A mesenteric hematoma was noted, and the arterial bleed was controlled temporarily with a clamp. Through and through penetrating injuries were found in the small bowel, about 30 cm proximal to the ligament of Treitz and involving about 20 cm of the bowel. Small bowel and associate mesentery resection was performed, and about 20 cm of small bowel was resected (Figure 1). The primary anastomosis was performed in a side-to-side isoperistaltic fashion.

At this point, the patient was hemodynamically stable, no active bleeding was noted, and there was a concern of possible duodenal injury or duodenal hematoma. The surgeon decided to perform an endoscopy rather than Kocherize the duodenum which would increase the operative time and could result in disrupting a non-expanding tamponade if present. A gastrointestinal consult was called into the operating room.

Following initial decompression of the stomach with a nasogastric tube (NGT), a gastroscope was inserted orally upon replacement of the NGT to enable visual monitoring of the small bowel interior. At the junction of the third/fourth part of the duodenum, a through and through injury was identified. No expanding retroperitoneal hematomas were noted in this zone. Given the difficult location of the injury, the controlled scenario with a stable patient, and an injury involving less than 40% of the bowel circumference, a decision was made to perform a primary repair. The repair was reinforced with an omental patch and a Blake drain left in the area of repair near the third portion of the duodenum/pancreas.

The patient was admitted to the critical care unit CCU postoperatively and received four additional units of PRBCs, four units of plasma, and one unit of platelets. The patient responded well to the transfusion, with hemoglobin stabilizing at 8.6 and platelets stabilizing at 485. An upper gastrointestinal series was done to rule out any leak or bowel stenosis on postoperative day six which showed no leak or obstruction. Computed tomography of the abdomen and pelvis was done on postoperative day 10 which showed no ileus or stenosis in the repaired area. The NGT was removed and the patient was started on clears on postoperative day 11. The patient remained hemodynamically stable and was discharged on postoperative day 17.

## Discussion

In the specialized context of trauma-induced duodenal hematoma, an intraoperative EGD can be increasingly helpful for rapidly identifying retroperitoneal bowel injuries without the need for the Kocher maneuver [1,2]. Duodenal hematoma, a consequence of trauma, poses unique challenges owing to the intricate proximity of the duodenum to surrounding structures. This proximity amplifies the vulnerability to concurrent retroperitoneal bowel injuries, accentuating the complexity of the clinical scenario [3]. This discussion intricately explores the pivotal role played by intraoperative EGD in both the precise diagnosis and effective management of retroperitoneal bowel injuries within the specific context of duodenal hematoma. The intricacies of this scenario highlight the advantages of leveraging the capabilities of EGD to navigate challenges associated with the diagnosis and management of retroperitoneal bowel injuries arising in tandem with duodenal hematoma.

Following trauma, the emergence of duodenal hematoma initiates a complex interplay, exerting significant pressure on neighboring retroperitoneal structures [3]. This cascade of pressure possesses the potential to induce injuries that might not manifest immediately, thereby introducing an additional layer of intricacy to the clinical scenario. Intraoperative availability of an experienced surgical team proficient in performing intraoperative EGD, or the availability of a gastrointestinal team, can greatly enhance the management of abdominal blunt or penetrating trauma during exploratory laparotomy. Rapid and effective execution of EGD in such scenarios holds significant value, as it enables the prompt identification of duodenal injuries without necessitating the time-consuming Kocherization maneuver. This approach is most beneficial in controlled environments where the necessary equipment and skilled personnel are readily accessible and responsive. The real-time visualization capabilities of the endoscope play a pivotal role in the comprehensive assessment of the gastrointestinal tract, with a specific focus on the duodenum and the contiguous retroperitoneal space. This nuanced exploration empowers surgeons to meticulously scrutinize these areas for subtle indicators of injury, perforation, or any trauma-related damage that could be obscured by the presence of the duodenal hematoma [4]. In patients with a history of extensive abdominal surgeries, employing EGD to visualize the duodenum may offer a more efficient alternative to the time-consuming Kocher maneuver. This approach is particularly advantageous in cases where prior surgical interventions have rendered the traditional Kocherization technique prolonged and challenging. The immediate insights provided by intraoperative EGD emerge as a crucial asset, guiding the surgical team in the identification and prompt addressing of potential retroperitoneal injuries. This ensures a thorough and targeted approach to trauma management, where the dynamic capabilities of EGD contribute significantly to optimal patient outcomes in the aftermath of duodenal hematoma-induced trauma [5].

An inherent advantage of integrating intraoperative EGD within the realm of duodenal hematoma lies in its distinctive capacity to navigate anatomically challenging areas, addressing potential limitations encountered in conventional surgical exploration [4]. The presence of hematoma significantly hampers the visual field, presenting challenges in the identification of potential injuries. However, the navigational prowess of the endoscope serves as a solution to this predicament by offering a flexible and precise tool. This facilitates an in-depth exploration of the retroperitoneal space, enabling a comprehensive assessment of potential bowel injuries that may be concealed or distorted by the hematoma [4]. The dynamic maneuverability of the endoscope ensures that even the most challenging or hidden regions are thoroughly examined, thereby augmenting diagnostic precision in the complex presence of duodenal hematoma [5]. This integration of EGD’s navigational capabilities enhances the surgeon’s ability to uncover obscured details and contributes to a more thorough evaluation of potential retroperitoneal injuries associated with duodenal hematoma, ultimately optimizing diagnostic accuracy and precision [5].

In situations where suspicion arises regarding retroperitoneal bowel injury in the context of duodenal hematoma, the incorporation of intraoperative EGD emerges as a pivotal and instrumental diagnostic and therapeutic tool [1,2]. This sophisticated instrument facilitates a meticulous examination of the bowel wall, a particularly critical aspect when the hematoma exerts compressive forces or induces distortions in the duodenum, thereby increasing its vulnerability to injury [3]. The immediate and detailed visualization provided by EGD becomes a guiding beacon for the surgical team, directing their attention to specific areas that necessitate intervention [5]. Integrating EGD into the surgical protocol not only aids in promptly identifying retroperitoneal injuries but also guides subsequent therapeutic interventions, ultimately enhancing patient outcomes in duodenal hematoma-induced trauma [1,2].

Furthermore, the inherent real-time capabilities of intraoperative EGD play a pivotal role in facilitating dynamic assessments throughout the entirety of the surgical procedure. This feature becomes particularly crucial in the specific context of duodenal hematoma, where the extent of injury may evolve or become more apparent as the surgery progresses. The ability to adapt and respond promptly to changes observed through EGD becomes a cornerstone for achieving better outcomes for the patient. This dynamic adaptability ensures that any retroperitoneal bowel injuries are not only promptly identified but also addressed in a timely and effective manner. The continuous real-time feedback provided by EGD empowers the surgical team to make informed decisions during the course of the procedure, contributing to an enhanced and tailored approach to managing retroperitoneal injuries associated with duodenal hematoma.

## Conclusions

Intraoperative EGD can play a pivotal role in identifying retroperitoneal bowel injury caused by trauma in the specific setting of duodenal hematoma. Its ability to provide real-time visualization, explore challenging areas, and guide surgical intervention without the need for a Kocher maneuver makes it a very advantageous tool in ensuring a thorough and targeted approach to managing retroperitoneal injuries in the presence of duodenal hematoma. This underscores the importance of integrating intraoperative EGD into surgical planning for trauma cases involving duodenal injuries, ultimately enhancing patient outcomes. Multidisciplinary collaboration can provide the opportunity to minimize risk for intraoperative EGD yet maximize utility in localizing unknown gastrointestinal bleeds during a challenging abdominal surgery.
